# Emerging Role of Mesenchymal Stromal Cell-Derived Extracellular Vesicles in Pathogenesis of Haematological Malignancies

**DOI:** 10.1155/2019/6854080

**Published:** 2019-06-02

**Authors:** Juçara Gastaldi Cominal, Maira da Costa Cacemiro, Belinda Pinto-Simões, Hans-Jochem Kolb, Kelen Cristina Ribeiro Malmegrim, Fabíola Attié de Castro

**Affiliations:** ^1^Graduate Program on Biosciences Applied to Pharmacy, School of Pharmaceutical Sciences of Ribeirão Preto, University of São Paulo, Ribeirão Preto, São Paulo, Brazil; ^2^Center for Cell-Based Therapy, Regional Blood Center of Ribeirão Preto, University of São Paulo, Ribeirão Preto, São Paulo, Brazil; ^3^Department of Internal Medicine, Ribeirão Preto Medical School, University of São Paulo, Ribeirão Preto, São Paulo, Brazil; ^4^Kolb Consulting UG München, Helmhotz Centre Muenchen & Univ Munich; Techn. Univ., Munich, Germany; ^5^Department of Clinical Analysis, Toxicology, and Food Sciences, School of Pharmaceutical Sciences of Ribeirão Preto, University of São Paulo, Ribeirão Preto, São Paulo, Brazil

## Abstract

Homoeostasis of bone marrow microenvironment depends on a precise balance between cell proliferation and death, which is supported by the cellular-extracellular matrix crosstalk. Multipotent mesenchymal stromal cells (MSC) are the key elements to provide the specialized bone marrow microenvironment by supporting, maintaining, and regulating the functions and fate of haematopoietic stem cells. Despite the great potential of MSC for cell therapy in several diseases due to their regenerative, immunomodulatory, and anti-inflammatory properties, they can also contribute to modulate tumor microenvironment. The extracellular vesicles that comprise exosomes and microvesicles are important mediators of intercellular communication due to their ability to change phenotype and physiology of different cell types. These vesicles may interact not only with neighbouring cells but also with cells from distant tissues to either maintain tissue homoeostasis or participate in disease pathogenesis. This review focuses on the current knowledge about the physiological role of MSC-extracellular vesicles, as well as their deregulation in haematological malignancies and their potential applications as biomarkers for diagnosis, progression, and treatment monitoring of such diseases.

## 1. Multipotent Mesenchymal Stromal Cells

**Figure 1:**
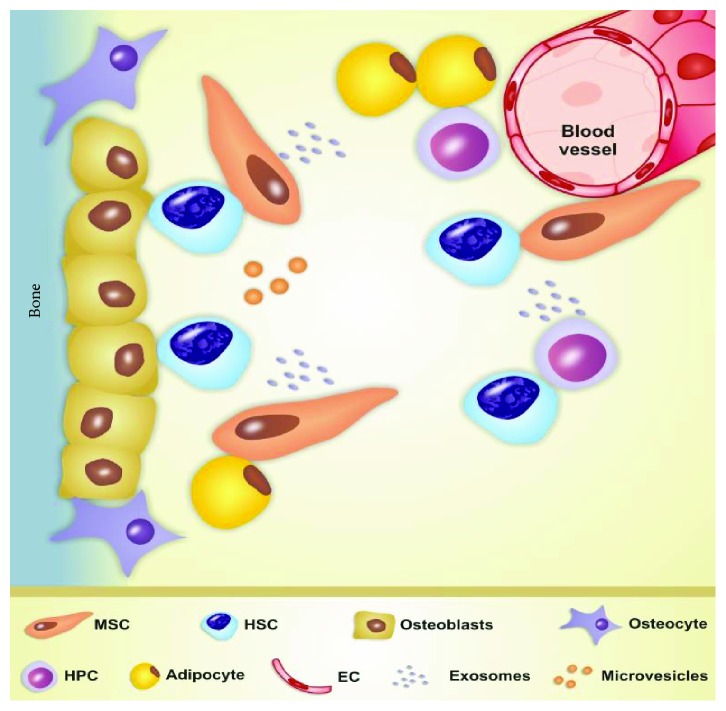
Schematic representation of the bone marrow (BM) microenvironment architecture and BM resident cell crosstalk via extracellular vesicles (exosomes and microvesicles) released from multipotent mesenchymal stromal cells (MSC). EC: endothelial cells; HPC: haematopoietic progenitor cells; HSC: haematopoietic stem cells.

Multipotent mesenchymal stromal cells (MSC), also known as mesenchymal stem cells or mesenchymal stromal cells, were described in the 1960s as a population of nonhaematopoietic cells of bone marrow (BM) microenvironment that support the haematopoiesis process [1, 2]. BM microenvironment is a very dynamic and integrated space composed of extracellular matrix, haematopoietic stem cells (HSC), haematopoietic progenitor cells, endothelial cells, and stromal cells including MSC, osteoblasts, osteoclasts, and adipocytes [3, 4]. MSC provide this specialized microenvironment known as the haematopoietic niche, which supports, maintains, and regulates the properties of HSC. Optimal conditions for HSC development depend on the existence of a preserved BM tissue architecture and BM resident cell crosstalk (Figure 1) [5, 6].

The interaction among HSC, MSC, and other cell types from BM microenvironment protects HSC from apoptotic and differentiation stimuli, keeping them quiescent and promoting self-renewal of the HSC pool [7, 8]. Secretion of interleukin- (IL-) 6, stem cell factor (SCF), and leukaemia inhibitory factor by MSC also supports haematopoiesis [9].

MSC have been isolated from perivascular space, adipose tissue, dental pulp, placenta, synovial tissue, and umbilical cord [2]. The multipotency of MSC enables them to differentiate *in vivo* into several mesoderm lineages including chondrocytes, osteocytes, and adipocytes [7, 8]. *Ex vivo* experiments also revealed that MSC are capable of transdifferentiating into nonmesodermal cell types such as neuroectoderm and endoderm lineages [7, 10].

The minimum criteria for MSC definition established by the International Society for Cellular Therapy in 2006 rely on their (i) ability to be plastic-adherent cells; (ii) multipotent potential to differentiate into osteocytes, adipocytes, and chondrocytes when cultured *in vitro* under specific conditions; and (iii) expression of the markers CD73, CD90, and CD105 and lack of CD45, CD34, CD14, CD19, and human leucocyte antigen DR (HLA-DR) expression [11].

MSC produce many types of bioactive molecules: (i) adhesion molecules, such as vascular cellular adhesion molecule-1 (VCAM-1), intercellular adhesion molecule-1 (ICAM-1), and activated leucocyte cell adhesion molecule (ALCAM); (ii) growth factors, such as SCF, transforming growth factor beta (TGF-*β*), epidermal growth factor (EGF), granulocyte-macrophage colony-stimulating factor (GM-CSF), and hepatocyte growth factor (HGF); (iii) cytokines, such as the interleukins IL-1*α*, IL-1*β*, IL-6, IL-7, and IL-8; (iv) angiogenic factors, such as vascular endothelial growth factor (VEGF) and platelet-derived growth factor (PDGF); and (v) immunomodulatory molecules, such as prostaglandin E2 (PGE_2_), human leucocyte antigen G (HLA-G), and indoleamine 2,3-dioxygenase (IDO) [12–15]. All these molecules account for the paracrine effects of MSC on neighbouring cells [16–18]. The production of such a wide array of bioactive molecules is ideal for MSC-based cellular therapies.

The rationale of MSC infusion to treat patients with immunological diseases is their ability to suppress or control exacerbated immune responses by inhibiting immune cell proliferation, inducing regulatory T/B lymphocyte proliferation, and favouring dendritic cell maturation through the secretion of immunosuppressive molecules and direct cell-to-cell contact [18–22]. The therapeutic use of MSC in regenerative medicine relies on their migration to injured tissues and promotion of endogenous regeneration by supporting the growth and differentiation of stem and progenitor resident cells, as well as by releasing proangiogenic, anti-inflammatory, chemotactic, and antiscarring factors [22–27].

However, MSC also participate in the tumorigenesis process of haematological malignancies and some solid cancers, such as adenocarcinomas, breast cancer, neuroblastoma, and osteosarcoma. MSC contribute to microenvironment malignant transformation and maintenance; i.e., they favour tumor cell growth, survival, and migration [5, 28]. On the other hand, MSC exert antitumor effect by inhibiting tumor cell proliferation, activating antitumor immune response, restricting tumor progression, and migrating to the tumor niche to promote tissue regeneration [29, 30].

In this sense, scientists and clinicians have considered MSC as a therapeutic agent and also as a therapeutic target due to their participation in the modification of BM microenvironment and chemoresistance in malignant diseases.

## 2. Extracellular Vesicles

Extracellular vesicles (EV) are a heterogeneous group of nanosized particles derived from cell membranes that are classified according to their biogenesis, size, molecular cargo, and membrane markers [5, 31–33]. Multiple names are used to identify the EV subtypes, being exosomes and microvesicles (MV) the two major classes [31].

Exosomes are nanostructures (30-150 nm size) of endocytic origin that are generated in the intraluminal vesicles of multivesicular bodies through a ceramide-dependent process and delivered to the extracellular space via exocytosis [31, 34]. MV, also called ectosomes or exosome-like vesicles, are 150-1000 nm vesicles released by budding of the plasma membrane with cytoskeleton involvement [34, 35]. Despite all the efforts to characterize EV, it is still a challenge to discriminate exosomes from MV because they share many similarities, including size range and expression of the protein markers CD63, CD81, and CD9 [31, 35, 36].

EV can be secreted by all eukaryotic cells and isolated from serum, blood, saliva, urine, breast milk, and semen [35]. They are key mediators of intercellular communication processes due to their ability to change the phenotype and physiology of neighbouring and long-distance cells [35, 37–39]. Once in the extracellular space, EV interact with many target cells through surface receptors, activate signalling pathways, and can be internalized by these cells via endocytosis, phagocytosis, pinocytosis, or membrane fusion [40, 41].

EV cargo comprises proteins, soluble factors, microRNAs (miRNAs), messenger RNA (mRNA), and DNA and varies according to cellular origin and biogenesis; i.e., it can reflect the molecular and functional characteristics of their parental cells [36, 42]. The biological effects of EV depend not only on their content but also on the functional and metabolic states of recipient cells [36]. Thus, EV may play a dual role by either mediating regulation of tissue homoeostasis or inducing a pathological process [6].

## 3. MSC-EV: Physiological Cell-to-Cell Communication

**Figure 2:**
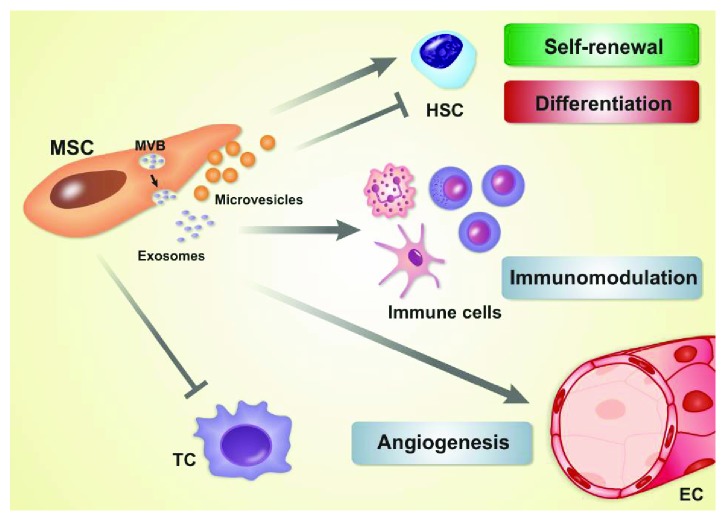
Physiological role of extracellular vesicles derived from multipotent mesenchymal stromal cells (MSC-EV) in bone marrow microenvironment. Exosomes are generated in the intraluminal vesicles of multivesicular bodies (MVB) and delivered to the extracellular space via exocytosis. Microvesicles are released by budding of the plasma membrane. MSC-EV regulate (induce) self-renewal and inhibit differentiation of haematopoietic stem cells (HSC) and exert immunomodulatory action by activating neutrophils and inhibiting proliferation of dendritic cells, NK cells, B-cells, and T-cells. MSC-EV activate angiogenesis in endothelial cells (EC) but inhibit angiogenesis in tumor cells (TC). MSC: multipotent mesenchymal stromal cells.

MSC intercellular communication can be mediated by EV secretion, exosomes, chemokines, cytokines, growth factors, structural protein components, metabolites, notch signalling, and gap junctional intercellular communication [42]. The secreted EV act as a signalling complex capable of stimulating target cells and modulating angiogenesis, HSC development, BM microenvironment, and immune system function (Figure 2) [43–55]. Characterization of MSC-EV revealed the presence of the transmembrane proteins CD107, CD63, CD9, and CD81 and the surface markers CD105, CD44, CD73, and CD29 [56–58].

MSC-EV may exert both pro- and antiangiogenic activities [43–47]. EV released from umbilical cord MSC induce angiogenesis and MSC migration and proliferation *in vitro* through the Wnt/*β*-catenin pathway; they also promote nuclear translocation of *β*-catenin, lower expression of E-cadherin, and augment expression of proliferating cell nuclear antigen, *β*-catenin, cyclin D3, and N-cadherin [43]. Interestingly, under hypoxic conditions, umbilical cord MSC produce EV rich in VEGF, VEGF receptor 2 (VEGFR2), monocyte chemoattractant protein-1 (MCP-1), angiogenin, IL-6, Tie-2/TEK receptor tyrosine kinase, and insulin-like growth factor (IGF) [44]. The NF-*κ*B signalling pathway is activated in EV-mediated angiogenesis, as evidenced by a comprehensive proteomic profiling of MSC-EV [45]. In addition, MSC-EV act on endothelial cells through miRNA transfer of proangiogenic miR-424, miR-30c, and miR-30b [46].

Paradoxically, murine BM-MSC-derived exosomes downmodulate VEGF expression and inhibit angiogenesis in breast cancer cell lines [47]. Exosomal miR-16 partially accounts for VEGF downmodulation *in vitro* and angiogenesis impairment *in vivo* [47].

MSC-EV contribute to HSC development by exerting haematopoiesis-supporting effects [48]. In a coculture *in vitro* system, the MSC-EV increase the CD34^+^ cord blood cell proliferation rate, upregulate *β*-catenin expression, and elevate the frequency of early haematopoietic precursor cells [48]. Although literature reports are controversial, the Wnt signalling pathway is considered as one of the essential pathways that regulate HSC by increasing *β*-catenin expression, as well as enhancing self-renewal and inhibiting differentiation of HSC [59]. Surprisingly, epigenetic analyses have revealed that (i) MSC-EV enriched with several microRNAs participate in the regulation of haematopoiesis and (ii) their predicted target genes are associated with the Wnt/*β*-catenin pathway inhibition and are expressed in CD34^+^ cord blood cells [48].

Recent studies have reported that BM-MSC-EV may play a role in BM microenvironment homoeostasis and maintenance [49, 50]. MV from normal BM-MSC contain proteins that act on cell proliferation, adhesion, migration, and morphogenesis, as revealed by proteomic analyses [49]. Normal BM-MSC release exosome-like EV with specific miRNAs; among which miR-143, miR-10b, miR-22, miR-486, and miR-21 are the most abundant ones. miR-143 has immunomodulatory functions, miR-10b and miR-22 regulate MSC differentiation, miR-486 promotes MSC survival and regulates their angiogenic activity, and miR-21 regulates cell cycle progression, proliferation, and angiogenesis [50].

MSC-EV also exert immunomodulatory activity: they impair dendritic cell maturation, activate neutrophils, inhibit NK cell proliferation and activation, suppress B- and T-cell proliferation, and increase regulatory T-cell population. The last effect is associated with high levels of IL-10-rich vesicles [51–55]. In summary, MSC-EV can induce either immune activation or suppression response depending on the target cell type and conditions of the surrounding milieu.

## 4. MSC-EV Contribution for Pathogenesis of Haematological Malignancies

MSC modulate tumor microenvironment in haematological malignancies [5, 60]. Haematological malignancies associated with malignant haematopoietic stem cells, such as myelodysplastic syndromes (MDS), myeloproliferative neoplasms (MPN), multiple myeloma (MM), and acute myeloid leukaemia (AML), depend on a favourable microenvironment with key protumor stimuli that provide conditions for tumor cell proliferation and survival [5].

The tumor microenvironment acts as the BM microenvironment under physiological conditions and regulates the malignant haematopoietic stem cell quiescence, self-renewal, and migration; MSC are the key cellular elements in this context [5]. Haematopoietic tumor cells mediate functional changes in stromal cells that may produce a protumor microenvironment and lead to disease onset or progression. MSC from MDS patients and healthy donors bear different transcriptome profiles, and the former is rich in proinflammatory and cellular stress genes [61].

Defective haematopoiesis in MDS patients contributes to the disease pathogenesis. In these patients, MSC IL-6 and IL-8 transcripts are upregulated and IL-6 and IL-8 inhibit haematopoiesis by downregulating the niche factors CXCL12 (*C-X-C motif chemokine ligand 12*), angiopoietin 1, and KIT ligand [61]. MSC from MPN, AML, and MDS patients exhibit functional and cytogenetic abnormalities that contribute to leukaemogenesis, while MSC from MPN patients also have reduced potential to support haematopoiesis *in vitro* [62, 63].

Considering that modifications on BM microenvironment are crucial to MM development, therapeutic-targeted deregulation of signalling between tumor and stromal cells has been successfully used in MM treatment [64]. MM cell survival, disease progression, and drug resistance are associated with alterations in MSC, including augmented gene expression of angiogenic and growth factors (such as CD40/40L, VCAM-1, ICAM-1, LFA-3 (*lymphocyte function-associated antigen-3*), and HO-1 (*heme oxygenase 1*), and immunomodulation of cytokines (increased IL-6 and reduced IL-10) [65, 66, 69]. MSC-induced drug resistance also occurs in acute lymphocytic leukaemia (ALL) and AML [67, 68, 70].

In contrast to the variety of aforementioned reports on MSC alterations in haematological malignancies [5, 60–69], the studies on the relationship between MSC-EV and pathogenesis of haematological malignancies are scarce. Some reports have discussed the participation of MSC-EV in other cancer types, including liver, lung, ovary, gastric, and breast cancers [71–75].

**Figure 3:**
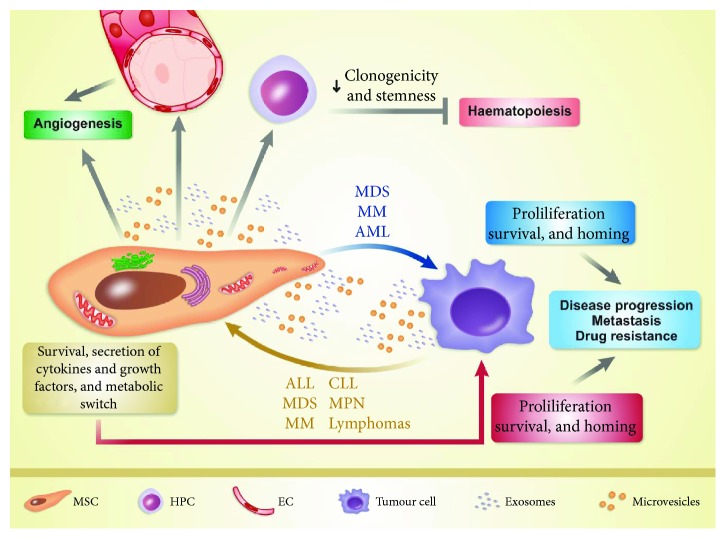
Interaction between multipotent mesenchymal stromal cells (MSC) and tumor cells in haematological malignancies. In acute myeloid leukaemia (AML), multiple myeloma (MM), and myelodysplastic syndromes (MDS), extracellular vesicles (EV) derived from multipotent mesenchymal stromal cells (MSC-EV) promote tumor cell proliferation, survival, and homing, leading to disease progression, metastasis, and drug resistance. MSC-EV induce direct or indirect angiogenesis and modulate clonogenicity and stemness of haematopoietic progenitor cells (HPC). Tumor-EV influence bone marrow-derived MSC (BM-MSC) by enhancing their survival and secretion of multiple cytokines and growth factors and promoting the metabolic switch. These alterations promote protumoral properties in BM-MSC, which in turn supply essential factors to tumor cells and improve their proliferation, survival, and homing. This bidirectional crosstalk also occurs in acute lymphocytic leukaemia (ALL), chronic lymphocytic leukaemia (CLL), lymphomas, multiple myeloma (MM), myelodysplastic syndromes (MDS), and myeloproliferative neoplasms (MPN). EC: endothelial cells.

The present review discusses the literature findings about BM-MSC-EV crosstalk between the haematopoietic niche and tumor cells in haematological malignancies (Figure 3), stratified by disease category.

**Figure 4:**
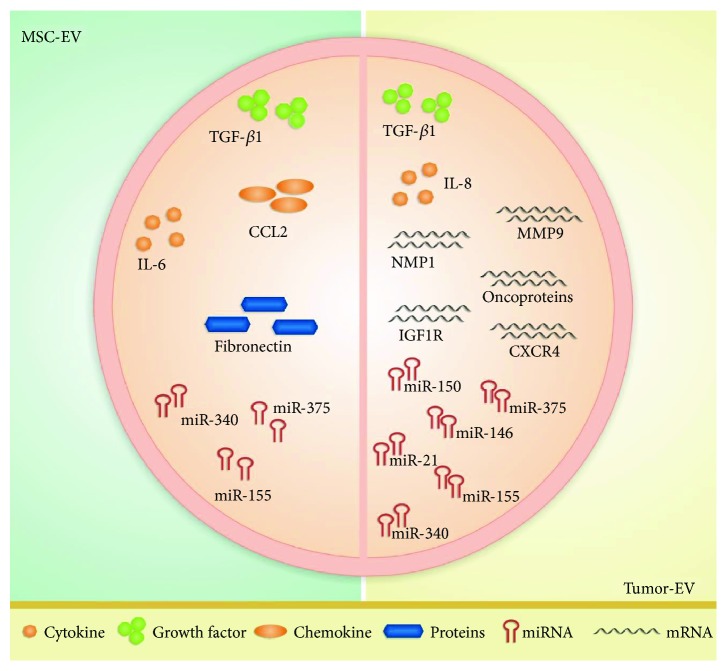
Differences between cargos of extracellular vesicles (EV) derived from multipotent mesenchymal stromal cells (MSC-EV) and tumor-EV in haematological malignancies. MSC-EV from patients with acute myeloid leukaemia, multiple myeloma, and myelodysplastic syndromes are enriched with TGF-*β*1, CCL2, IL-6, fibronectin, miR-375, miR-340, and miR-155. In several haematological malignancies, tumor-EV cargo reveals high amounts of TGF-*β*1 and IL-8 and the microRNAs miR-21, miR-146, miR-150, miR-155, miR-340, and miR-375.

Some research groups have investigated the effects of BM-MSC in MM [76–79]. Exosomes released from MM patients' BM-MSC modulate disease progression *in vivo* by increasing the exosome-based delivery of IL-6, CCL2 (*C-C motif chemokine ligand 2*), and fibronectin and decreasing expression of the tumor suppressor miR-15a. The miR-15a is capable of inhibiting MM cell proliferation and inducing apoptosis, maintaining the disease in a stable state (Figure 4) [76].

MSC-EV participate in tumor cell homing *in vivo*, indicating that BM-MSC-EV play a role in metastasis. Mice with severe combined immunodeficiency simultaneously administered with the MM cell line MM.1S and primary MM BM-MSC-derived exosomes present higher tumor growth and metastasis rates than mice administered with MM.1S cells alone or in combination with normal BM-MSC-derived exosomes [76].

BM-MSC-derived exosomes from MM promote cell proliferation and increase cell survival by activating the AKT pathway and inhibiting the p38, p53, and c-Jun N-terminal kinase (JNK) pathways in a mouse model [77]. Such BM-MSC-derived exosomes may lead to MM cell line resistance to the proteasome inhibitor bortezomib, which seems to be associated with impairment of apoptosis mediated by increased Bcl-2 expression and decreased levels of cleaved poly-(ADP-ribose) polymerase (PARP), caspase-9, and caspase-3 proteins [77].

A study that investigated how BM-MSC-MV from normal subjects and MM patients affect the phenotype, translation initiation, and MAPK signalling in five MM cell lines (U266, ARP-1, MM.1S, OPM-2, and RPMI 8226) revealed that tumorigenesis is associated with enhanced levels of the eukaryotic translation initiation factors 4E and gamma-1 (eIF4E and eIF4GI, respectively), which upregulate the translation of crucial oncogenes [78].

Treatment of MM cell lines with normal BM-MSC-MV elicits a rapid MAPK pathway activation followed by a decrease in viability, proliferation, migration/invasion potential, and translation initiation process, while treatment with BM-MSC-MV from MM patients induces a rapid and persistent MAPK activation that exerts the opposite effect on the aforementioned parameters [78].

A study on the effect of BM-MSC exosomes from old and young healthy donors using an MM *in vivo* hypoxic bone marrow model [79] evidenced that (i) young BM-MSC exosomal miR-340 inhibits tumor angiogenesis through the hepatocyte growth factor/c-MET pathway more strongly than old BM-MSC exosomes (Figure 4) and (ii) old BM-MSC hold weaker immunomodulatory potential and functional changes in genes related to developmental processes, cell adhesion, and proliferation. Such age-associated modifications that impair the antitumor properties of BM-MSC may be related to cancer, especially because most of the cancer processes are age-related [79].

BM-MSC-MV from low-risk MDS patients promote modifications in CD34^+^ haematopoietic progenitor cells. Treatment of these cells with MV overexpressing miR-10a and miR-15a upregulates the tumor protein p53 proto-oncogene and downregulates MDM2, a p53 regulator [80]. BM-MSC-MV from MDS patients, but not from healthy individuals, are capable of altering CD34^+^ cell behaviour by increasing their survival and clonogenic capacity without altering their immunophenotype and differentiation potential [80].

BM-MSC release exosomes rich in TGF-*β*1, miR-155, and miR-375 (Figure 4) [81]—the last two are used as markers of risk disease status in AML. These exosome-based effects significantly correlate with AML cell resistance to tyrosine kinase inhibitors [81]. Future studies are necessary to elucidate the interactions between BM-MSC exosomes and AML niche associated with chemoresistance [81], since this knowledge can help to develop novel therapeutic approaches targeting BM-MSC to prevent drug resistance and minimal residual disease.

## 5. Tumor-EV from Haematological Malignancies Influence BM-MSC

Several reports have demonstrated the existence of a crosstalk between tumor cell EV and BM-MSC in haematological malignancies (Figure 3) [82–95]. Here, we summarize some remarkable studies, stratified by influence/relevance of the major process: (i) angiogenesis and cell proliferation [82–86]; (ii) BM microenvironment modifications [85, 87–89]; (iii) beneficial effects on tumor cells [90–92]; and (iv) metastasis, disease progression, drug resistance, and disease diagnosis/monitoring [85, 89, 93–95].

Enhancement of angiogenesis is a process shared by chronic myeloid leukaemia (CML), chronic lymphocytic leukaemia (CLL), ALL, AML, MM, MDS, MPN, and lymphomas that have been associated with severity and progression of these haematological diseases [96, 97]. Different mechanisms have been proposed to explain the role of tumor-EV in angiogenesis [98], especially those that affect the MSC properties and haematopoietic niche transformation into haematological malignancies [82, 83].

Plasma MV from CLL patients seem to activate the AKT/mTOR pathway in BM-MSC from CLL patients and elicit VEGF and hypoxia-inducible factor 1 (HIF-1) production, which thereby increase the proangiogenic potential. These effects contribute to CLL cell survival and resistance to rituximab/alemtuzumab [82]. In addition, plasma MV upregulate the cell cycle regulator cyclin D1 and MYC protein expression in BM-MSC from CLL patients, increasing their proliferation potential and survival; however, plasma MV from CLL patients do not exert the same effect in BM-MSC from healthy donors [82].

The action of IL-8 secreted from BM-MSC may explain the exacerbated angiogenesis in CML [83]. Exosomes derived from the CML cell line LAMA84 trigger IL-8 production in the MSC cell line HS5, which in turn contributes to leukaemogenesis by enhancing survival, proliferation, and migration of LAMA84 cells *in vitro* [83].

In addition to angiogenesis enhancement that tumor-EV promote in CML, MV from the CML cell line K562 may transfer the *BCR-ABL1* mRNA to normal BM-MSC and induce *BCR-ABL1* ectopic expression, leading to exacerbated MSC proliferation and TGF-*β*1 secretion, without cytogenetic abnormality (Figure 4) [84].

Exosomes from CLL patients strongly modulate niche stromal cells and favour disease progression by enhancing cellular angiogenesis, proliferation, migration, and cytoskeleton remodelling [85]. These exosomes are associated with alterations in normal BM-MSC, such as induction of the cancer-associated fibroblast phenotype, characterized by increased cell proliferation and production of cytokines and chemokines—including IL-8, B-cell activating factor (BAFF), C-X-C motif chemokine ligand 1 (CXCL1), leukaemia inhibitory factor, IL-6, IL-34, C-C motif chemokine ligands 2 and 5 (CCL2 and CCL5, respectively)—as well as of the migration/invasion factors claudin 1 (CLDN-1), epithelial stromal interaction 1 (EPSTI-1), ICAM-1, and matrix metalloproteinase 1 (MMP-1) [85]. In solid tumors, this phenotype is recognized as a malignant promoter that contributes to metastasis [85].

Human T-cell lymphotropic virus type I (HTLV-I) infection causes adult T-cell leukaemia/lymphoma (ATL) [86]. Leukaemogenesis depends not only on HTLV-I infection and cell transformation but also on stromal cell shift to protumoral environment [86]. Exosomes from ATL patients' primary cells and the cell lines C81 and Hut-102 carry miR-21, miR-155, and the viral oncoprotein Tax, reflecting the parental cell phenotyping (Figure 4) [86]. Primary BM-MSC from ATL patients are modulated by ATL patients' exosomes containing the Tax oncoprotein responsible for NF-*κ*B pathway activation, which increases MSC proliferation [86]. In addition, ATL progression may be related to reduction of MSC stemness and improvement of angiogenesis due to the increased levels of VEGF, CXCR4, and MMP-9 [85].

AML primary cells and the cell lines HEL 92.1.7, HL-60, MOLM-14, and U937 release exosomes rich in mRNAs related to niche modulation, responsiveness to treatment, and disease prognosis (Figure 4) [87]. Exosome-mediated transfer of mRNA of IGF1R (*insulin-like growth factor 1 receptor*), MMP-9 (*matrix metalloproteinase 9*), NPM1 (*nuclear matrix protein 1*), CXCR4 (*C-X-C motif chemokine receptor 4*), and FLT3-ITD (*internal tandem duplication mutations in FLT3*) to both normal BM-MSC, murine OP9 cells, and human CD34^+^ cells is a mechanism by which exosomes from AML cells modulate haematopoiesis and niche microenvironment (Figure 4) [87].

Exosomes from AML primary cells influence the BM-MSC from AML patients by negatively modulating the transcription factors C-MYB, HOXA-9 (*homeobox A9*), and CEBP-*β* (*CCAAT/enhancer binding protein beta*) involved in haematopoiesis control [87]. Exosomes from AML patients also deregulate the BM-MSC functions due to exosome cargo, including miR-155, miR-375, and miR-150 (Figure 4) [87]. The miRNAs regulate cell proliferation and secretion of growth factors and cytokines and decrease CXCR4 expression, contributing to disease pathogenesis [87].

Large B-cell lymphoma cells harbour a mutation in *MyD88* (*myeloid differentiation primary response 88*) gene, a key signalling molecule that interacts with Toll-like and IL-1 receptors and sustains lymphoma cell survival. Lymphoma cells shed EV with mutated *MyD88* (Figure 4), which induces a proinflammatory BM microenvironment and causes BM niche deregulation and inefficient haematopoiesis [88].

Primary BM-MSC from MDS/AML patients, but not from healthy donors have decreased levels of the haematopoietic factors SCF and JAG1 (*Jagged 1*) [89]. The MSC alterations in MDS/AML patients can result from the exosome crosstalk between MDS/AML cells and BM-MSC. EV from patients' primary cells decrease *SCF* and *JAG1* expression in BM-MSC, in a coculture approach [89]. On the other hand, BM-MSC exosomes from primary MDS/AML patients modulate normal BM CD34^+^ progenitor/stem cells by reducing their clonogenicity and stemness [89].

It is well-known that tumor cells can switch their metabolism from oxidative phosphorylation to aerobic glycolysis in order to improve tumor cell survival. The metabolic switch stimulates the ALL blasts to release EV [90]. EV from the ALL cell lines SD1 and NALM6 induce the same metabolic alterations in the MSC cell line HS5, which in turn dampen their response to oxidative stress and mitochondrial respiration with an excess of lactate production. MSC release lactate into the extracellular fluid, which serves as an energetic vital source to ALL blasts [90].

Exosomes from MM cell lines stimulate protumoral properties in BM-MSC from healthy donors via miRNA transfer [91]. Overexpression of the cancer progression-associated miR-146a in MSC after incubation with exosomes from the MM cell lines RPMI 8226, OPM-2, LP-1, and U266 induces secretion of IL-6, CXCL1, IP-10 (*interferon gamma-induced protein 10*), and CCL5 and enhances tumor cell survival and migration [91]. Blockage of the notch pathway decreases secretion of these miRNA-linked cytokines that are essential for MM cells [91]. A previous study from Reagan et al. [92] revealed that primary BM-MSC from MM patients express high levels of miR-146a *in vivo* [92], demonstrating that tumor cells modulate BM-MSC cells.

The miR-let-7b and miR-18a contents of circulating exosomes also reveal an association with the MM patients' outcome, as progression-free survival and overall survival (Figure 4) [93]. Thus, the miR from exosomes or MV can be used in newly diagnosed MM patients as a biomarker for disease prognosis [93].

TGF-*β*1 levels in plasmatic exosomes from AML patients are associated with AML progression and resistance to treatment (Figure 4) [94]. The levels of exosomal proteins vary according to the AML patients' status: the TGF-*β*1 level is higher in AML patients at diagnosis and in patients resistant to therapy; patients who respond to chemotherapy present lower TGF-*β*1 levels than patients who are resistant to chemotherapy [94].

Increased levels of circulating EV rich in tumor-related antigens are detected in sera from patients with haematological malignancies, when compared with sera from healthy subjects [95]. The tumor-related antigens reported in circulating MV are CD38^+^ in MM, CD19^+^ in B-cell neoplasms (CLL and non-Hodgkin's lymphoma), CD13^+^ in myeloid neoplasms (AML, CML, and MDS), and CD30^+^ in Hodgkin's lymphoma [95]. High MV levels are associated with poor prognosis scores in CLL patients in advanced Rai stage, MM patients in international staging system 3, MDS patients in RAEB-2 stage (*refractory anaemia with excess blasts stage 2*), and in Hodgkin's lymphoma patients in stage 3B-4 [95]. Although the studies with circulating EV [93–95] did not identify the parental cells responsible for EV secretion, it is reasonable to hypothesize that both tumor-EV and MSC-EV exert this function. Hence, circulating EV-tracking can be a useful noninvasive biomarker for disease diagnosis and monitoring.

## 6. Conclusions and Future Directions

EV participate in cell-to-cell communication in the haematopoietic niche and play important roles in several physiological processes. MSC-EV contribute to disease pathogenesis by supporting the transformation of BM microenvironment into a protumoral niche in haematological malignancies, enhancing angiogenesis, and promoting optimal conditions for tumor cell survival, proliferation, migration, and drug resistance.

Despite all this knowledge, there are many challenges to overcome in the MSC-EV research field, including (1) to optimize the EV isolation and characterization methods to achieve accurate data replication; (2) to elucidate the biogenesis mechanisms underlying EV; (3) to understand how the interactions between EV and recipient cells occur, as well as how to modulate EV uptake; (4) to unravel the mechanisms that regulate EV release in health and disease conditions; and (5) to investigate the MSC-EV from sources other than BM and their involvement in haematological malignancies.

Elucidation of these issues and of the role that MSC-EV play in the physiopathology, progression, and drug resistance in haematological malignancies shall enable researchers to design effective therapies for neoplastic patients in a near future, using MSC as either a therapeutic target or a therapeutic agent.

Finally, the researchers should be aware of the advantages and disadvantages of MSC-EV and seek strategies for successfully translating EV biology research into an effective therapy for haematological diseases.

## Abbreviations

ALCAM: Activated leukocyte cell adhesion molecule
ALL: Acute lymphocytic leukaemia
AML: Acute myeloid leukaemia
ATL: Adult T-cell leukaemia/lymphoma
BM: Bone marrow
BM-MSC: Multipotent mesenchymal stromal cells from bone marrow
BM-MSC-EV: Extracellular vesicles from multipotent mesenchymal stromal cells from bone marrow
BM-MSC-MV: Microvesicles from multipotent mesenchymal stromal cells from bone marrow
CCL: C-C motif chemokine ligand
CD: Cluster of differentiation
CLL: Chronic lymphocytic leukaemia
CML: Chronic myeloid leukaemia
CXCL1: C-X-C motif chemokine ligand 1
CXCR4: C-X-C motif chemokine receptor 4
DNA: Deoxyribonucleic acid
EV: Extracellular vesicles
HSC: Haematopoietic stem cell
HTLV-I: Human T-cell lymphotropic virus type I
ICAM-1: Intercellular adhesion molecule 1
IDO: Indoleamine 2,3-dioxygenase
IL: Interleukin
JAG1: Jagged 1
MDS: Myelodysplastic syndromes
MM: Multiple myeloma
MMP: Matrix metalloproteinase
MPN: Myeloproliferative neoplasms
mRNA: Messenger RNA
miR/miRNAs: microRNAs
MSC: Multipotent mesenchymal stromal cells
MSC-EV: Extracellular vesicles from multipotent mesenchymal stromal cells
MV: Microvesicles
MyD88: Myeloid differentiation primary response 88
RNA: Ribonucleic acid
SCF: Stem cell factor
TGF-*β*: Transforming growth factor beta
VCAM-1: Vascular cellular adhesion molecule-1
VEGF: Vascular endothelial growth factor.
